# Breaking Down Barriers in Pursuit of Thrombolytic Perfection∗

**DOI:** 10.1016/j.jacadv.2023.100673

**Published:** 2023-10-24

**Authors:** Yevgeniy Brailovsky, Waqas Ullah

**Affiliations:** Division of Cardiology, Department of Internal Medicine, Thomas Jefferson University Hospital, Philadelphia, Pennsylvania, USA

**Keywords:** CTED/CTEPH, post thrombotic syndrome, pulmonary embolism, right ventricular failure, thrombolysis

**Figure:**
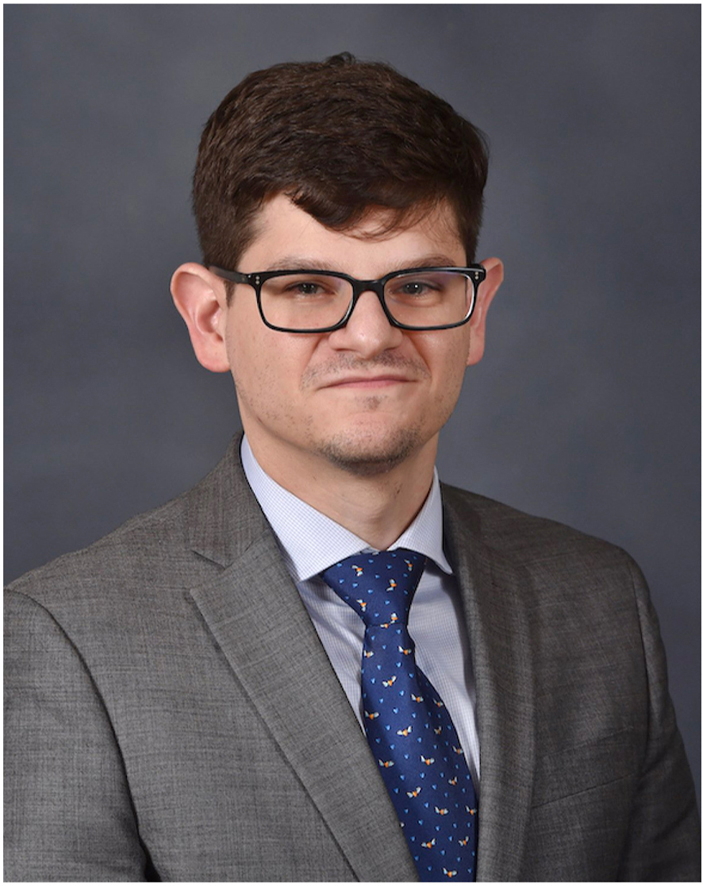

Patientswith acute pulmonary embolism (PE) are at increased risk of decompensation, mortality, and long-term risk of chronic thromboembolic disease (CTED) and chronic thromboembolic pulmonary hypertension (CTEPH). Current risk stratification models accurately identify patients at low risk of complications, who can be treated conservatively, and those at highest risk who need immediate intervention. However, these contemporary risk models struggle to categorize patients who are initially stable but may decompensate and need escalation of therapy.^1^ There has recently been an extraordinary expansion of catheter-based options for the treatment of acute PE, including standard catheter-directed thrombolysis (CDT) using multiside-hole infusion catheters or ultrasound-assisted thrombolysis (USAT) devices, small and large bore catheter-directed thrombectomy devices, and pharmaco-mechanical catheter directed thrombolysis (PM-CDT) with the Bashir catheter.^2^ Unlike other devices, the Bashir catheter consists of an expandable basket of 6 nitinol reinforced infusion limbs, which act to fragment the thrombus, create mini channels of blood flow, and increase the surface area for endogenous and exogenous thrombolysis to take effect.^2^ However, there has been a lack of large, randomized controlled trials (RCTs) and no head-to-head comparisons between different devices. As a result, there still exists an equipoise as to which patients need escalation of care, which catheter to choose, and which outcome measure to follow (Figure 1).

**Figure 1:**
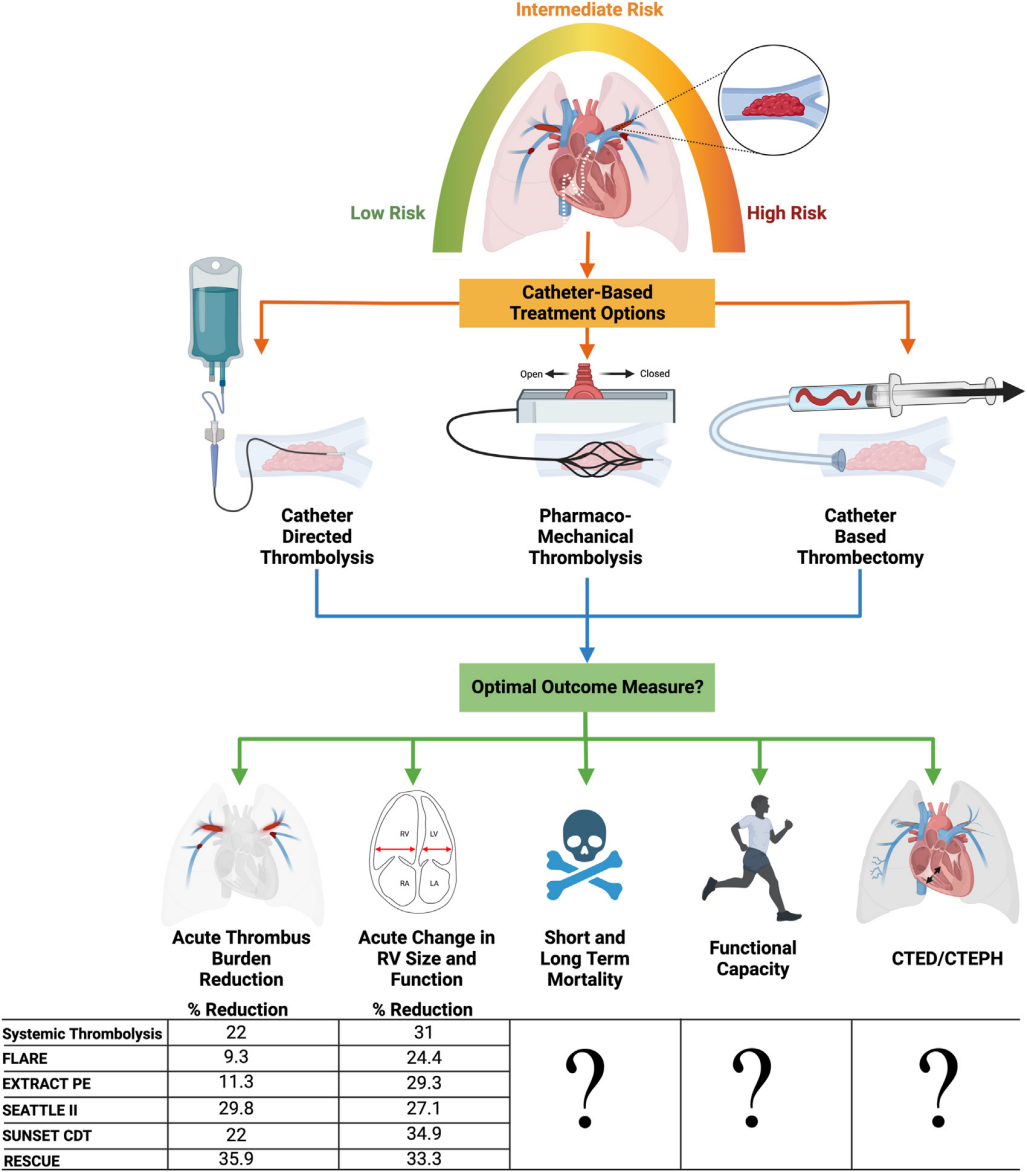
**Approaches to Pulmonary Embolism Managementand Outcome Measures** Figure created with BioRender.com.

In this issue of *JACC: Advances*, Bashir et al^2^ shed light on some of these unanswered questions, particularly the impact of PM-CDT on total and subtotal segmental and proximal branch pulmonary artery (PA) occlusions for treatment of acute PE.^2^ The authors demonstrate that at 48 hours post-PM-CDT, there was a decrease in segmental PA branches with total or subtotal occlusions (40.5% to >11.6%, *P* < 0.0001) and proximal PA branch total or subtotal occlusions (28.7% to >11.0%, *P* < 0.0001). Interestingly, these improvements were also seen in the branches that were remote from the location of the infusion basket.^2^ This may be a function of improved flow by clot fragmentation due to basket expansion and increased endogenous thrombolysis. However, these observations are speculative, and more studies are needed to elucidate this further.

Though many contemporary studies of catheter-based treatments of acute PE have also demonstrated a reduction in PA obstruction at 48 hours post-treatment, it appears that the magnitude of reduction in clot burden appears to be greatest with the use of PM-CDT as shown by Bashir et al (Figure 1). However, these findings should be interpreted with caution due to the lack of a control arm in the study, and heterogeneity in patient selection.

The current study also demonstrates a significant decrease in right ventricle/left ventricle (RV/LV) ratio, (1.66 ± 0.45 to >1.10 ± 0.24) 48 hours post-PM-CDT, consistent with other PE interventional trials (Figure 1).^2^ However, the novel finding of the current study is that the reduction in segmental artery occlusions correlated significantly with the magnitude of reduction in RV/LV ratio (correlation coefficient 0.287, *P* = 0.0026), whereas that in the proximal PA arteries did not (correlation coefficient 0.132, *P* = 0.173). This suggests that it is insufficient to simply reduce clot burden but, more importantly, to improve downstream occlusion and increase LV filling. This is also supported by prior work that demonstrated that decreased contrast transit from the PA to the left atrium is associated with poor cardiac index and worse outcomes.^3^ Additional studies are needed to investigate further the differential impact of various catheters on clot reduction in the proximal and distal vascular beds.

Residual pulmonary obstruction is an independent predictor of subsequent late complications, including recurrent venous thromboembolism and CTEPH, and that the impact of residual pulmonary obstruction increased according to the degree of the perfusion defects.^4^ In this context, it would be interesting to compare the long-term outcomes such as post-PE syndrome like CTED and CTEPH of patients treated with PM-CDT with those that treated with conventional catheters. PE-TRACT (Pulmonary Embolism - Thrombus Removal With Catheter-Directed Therapy) is a National Institutes of Health sponsored trial that will investigate the impact of CDT on long-term functional assessment in patients with acute PE.^5^

Furthermore, in a RCT (SUNSET sPE) comparing standard CDT to USAT, investigators failed to demonstrate a superiority of USAT over standard CDT with similar pulmonary arterial thrombus reduction using comparable mean lytic doses.^6^ Given that the cost of USAT catheters is significantly higher than standard multiside-hole catheters, this study raised concerns regarding their routine use for CDT. Similarly, although the Bashir catheter provides a unique design exposing a greater surface area to thrombolytic agents, future studies comparing standard CDT to the Bashir catheter would help justify the increased cost of this device and to see if lower lytic doses can be used.

It is important to note that some patients may have absolute contraindications to thrombolytics, which may prevent the use of CDT to treat acute PE, making mechanical thrombectomy the treatment of choice. Therefore, it is crucial to maintain percutaneous thrombectomy as part of our armamentarium and assess its safety and efficacy compared to anticoagulation alone and other catheter-based approaches. PEERLESS (A prospective, multicenter, randomized controlled trial of the FlowTriever System compared to Catheter-Directed Thrombolysis for use in the treatment of acute pulmonary embolism) is a RCT aimed to compare percutaneous thrombectomy with the FlowTriever system with CDT in hemodynamically stable patients with acute PE.^7^

The total r-tissue plasminogen activator (tPA) dose administered in this study was 7 mg in unilateral and 14 mg in bilateral PE patients, which is less than used in ULTIMA (Ultrasound Accelerated Thrombolysis of Pulmonary Embolism) trial (12 mg ×2), but more than low tPA dose of OPTALYSE (A Randomized Trial of the Optimum Duration of Acoustic Pulse Thrombolysis Procedure in Acute Intermediate-Risk Pulmonary Embolism) (4 mg ×2).^8^^,^^9^ It is interesting to see whether the expandable basket would allow lower tPA to be used, as the basket fragments the clot and increases the surface area for exogenous as well as endogenous thrombolytics to take effect. The endogenous fibrinolytic system is a complex framework with several key inducers (tPA, urokinase-type plasminogen activator) and inhibitors (plasminogen activator inhibitor-1, thrombin-activatable fibrinolysis inhibitor, and alpha-2-antiplasmin) of endogenous thrombolysis. During an episode of acute PE, the inhibitors of the endogenous fibrinolytic system are substantially dysregulated, which is associated with increased mortality.^10^ It would be of interest to evaluate the impact of PM-CDT on the markers of endogenous fibrinolytic system and its effect on patient outcomes.

It remains unclear what the optimal end points for interventional PE trials are. While overall survival appears to be an attractive measure, the fortunate truth is that mortality in the intermediate PE group remains low, and to demonstrate mortality benefit, we would need a rather large trial. Furthermore, a substantial proportion of patients with acute PE die from non-PE-related complications, such as malignancy, and it is unlikely that PE intervention alone would substantially alter overall survival. On the other hand, surrogate markers such as acute changes in RV size and function and clot burden are objective, easy to obtain, and change acutely with PE-related intervention. However, it is not clear how such markers relate to patient-reported outcomes or long-term mortality. There seems to be a delayed “catch-up” period of RV/LV ratio, even in patients treated conservatively, so longer term trials are needed. Each device offers its unique benefit over conservative management and potentially over other devices. It is of great interest to assess the impact of PE intervention on patient-centric outcomes measures, like quality of life, short- and long-term mortality, and incidence of CTED and CTEPH.

## Funding support and author disclosures

The author has reported that they have no relationships relevant to the contents of this paper to disclose.
